# Pre-Clinical In-Vitro Studies on Parameters Governing Immune Complex Formation

**DOI:** 10.3390/pharmaceutics14061254

**Published:** 2022-06-13

**Authors:** Marie Fichter, Gesa Richter, Alexander Bepperling, Paul Wassmann

**Affiliations:** 1 Novartis Institutes for BioMedical Research, Novartis Pharma AG, 4056 Basel, Switzerland; 2 TRD Biologics & CGT, Novartis, 83607 Holzkirchen, Germany

**Keywords:** immune complexes, protein complexes, anti-drug antibodies, immunogenicity, biotherapeutics, analytical assessment, aggregation, mass photometry, SEC-MALS, SV-AUC

## Abstract

The success of biotherapeutics is often challenged by the undesirable events of immunogenicity in patients, characterized by the formation of anti-drug antibodies (ADA). Under specific conditions, the ADAs recognizing the biotherapeutic can trigger the formation of immune complexes (ICs), followed by cascades of subsequent effects on various cell types. Hereby, the connection between the characteristics of ICs and their downstream impact is still not well understood. Factors governing the formation of ICs and the characteristics of these IC species were assessed systematically in vitro. Classic analytical methodologies such as SEC-MALS and SV-AUC, and the state-of-the-art technology mass photometry were applied for the characterization. The study demonstrates a clear interplay between (1) the absolute concentration of the involved components, (2) their molar ratios, (3) structural features of the biologic, (4) and of its endogenous target. This surrogate study design and the associated analytical tool-box is readily applicable to most biotherapeutics and provides valuable insights into mechanisms of IC formation prior to FIH studies. The applicability is versatile—from the detection of candidates with immunogenicity risks during developability assessment to evaluation of the impact of degraded or post-translationally modified biotherapeutics on the formation of ICs.

## 1. Introduction

Therapeutic proteins, also called biotherapeutics or biologics, are a successful class of drugs with yearly increasing numbers of approved new drugs [1] that have clear medical advantages such as high specificity and extended half-life. A main limitation of biotherapeutics is related to unwanted immune responses, which in some cases are of relevance for the safety of patients. Recent studies indicate the formation of patient-derived anti-drug antibodies for most biotherapeutics [2]. Only in rare and worst cases are severe and life-threatening adverse events the consequences, respectively [3]. The pharmaceutical industry is guided by health authorities to detect and monitor the immunogenicity of the biotherapeutic candidates [4,5] early on. Due to the differences between animal and human immune responses [6], the predictive value of animal studies to assess the incidence and impact of immunogenicity is limited, and certain aspects can only be assessed in detail during clinical studies. Besides the potential risks of immunogenicity to healthy volunteers or patients, the identification of immunogenic liabilities only during late stages of the drug development process may jeopardize the whole program. This is not only costly, but can lead to dramatic delays for awaited new medicines considering the long development times for biotherapeutics [7,8]. Therefore, an earlier insight into factors leading to immunogenicity of biotherapeutic candidates and their potential impact would be advantageous. The most suitable moment is during developability assessment, a stage dedicated to the selection of the superior candidate from a cohort of potential drug candidates against the same target, including manufacturability and molecular quality attributes [9,10]. An additional advantage is the possibility to eliminate detected immunogenic motifs via engineering without strongly delaying the program. Multiple immunogenicity prediction tools are available ranging from bioinformatics with the single focus on the amino acid sequence [11], to sophisticated in-vitro cell based analytical approaches like the MAPPS assay [12,13] or T cell based assays. These approaches are limited by the vast diversity and interdependency of factors that are of importance for the initiation of immunological responses. At the current state, such tools are not capable to predict the magnitude and impact of the immunological response(s) in humans.

As indicated by healthcare agencies [4,5], the complexity of the immunological response is driven primarily by drug product-related factors and further impacted by patient and disease-related factors. Drug product-related factors include the presence of neo-epitopes like non-human amino acid sequence stretches in the biotherapeutic and artificial protein constructs formed by protein engineering [14]; physical and chemical degradation and herewith associated stability of the drug material in biofluids [15]; the multimerization state of the biotherapeutic and its pharmacologic target in the body; “high” concentration of the biotherapeutic; especially in the case of mimicking a low-concentration endogenous protein; and many more [16].

The formation of ADAs may either have no effect, alter pharmacokinetics, or lead to neutralization of the biotherapeutic and reduce the efficacy of the drug [2]. Further, immune complexes (ICs) formed by the cross-linking of the biotherapeutic and the circulating ADAs will trigger additional responses in the immune cascade [16,17] and/or lead to faster clearance of the biotherapeutic observed in pharmacokinetics and pharmacodynamics studies [18].

Monitoring of free circulating ADAs in serum samples is critical during clinical trials for biotherapeutics, but immune complex formation is typically not monitored. Since factors such as the dynamic concentration of the biotherapeutic (clearance), dynamics of ADA concentrations, and the pharmacologic target (compartmentalization) are contributing to the formation of ICs, in-vitro testing platforms are best suited for the assessment of IC formation and characterization, as they are considering the impact of individual IC building blocks. At early research and development phases such analyses can be performed with surrogate ADAs, giving valuable insights into immune complex-forming processes.

The background of biofluids is impacting on the performance of physicochemical methods analyzing ICs, such as size-exclusion chromatography (SEC) and sedimentation velocity analytical ultracentrifugation (SV-AUC), leading to low reproducibility and inaccurate quantification. Pre-assessment of the analytical method performance using biological matrices or in-vitro generated ICs is required to prevent artifact-driven results.

The aim of our study was to set up an in-vitro IC characterization platform, which can be used as a tool for gaining mechanistic insights into attributes of relevance for IC formation, composition, and sizes. The following attributes were assessed: absolute concentration of the IC building blocks, molar ratio between the components, number of neo-epitopes recognized by ADAs, quality of the biotherapeutic material, impact of the pharmacologic target antigen on IC formation, and the impact of serum components. Additionally, we tested the suitability, performance, and limitations of a new physico-chemical method, i.e., mass photometry, and the classical methods SEC(-MALS), SV-AUC, and DLS to characterize ICs. Our data is demonstrating the impact of multiple factors modulating the characteristics of the ICs, whereby the molar ratio between the IC building blocks is shown to be the key driver for the dynamics of formed ICs, i.e., their relative amount and their sizes.

## 2. Materials and Methods

### 2.1. Material

10× phosphate-buffered saline (PBS, #P0195, Teknova, Hollister, CA, USA), di-sodium hydrogen phosphate/sodium dihydrogen phosphate (#106586/#106346, Merck, Darmstadt, Germany), sodium perchlorate monohydrate (#89152, Sigma, Buchs, Switzerland), thyroglobulin (#T9145, Sigma, Buchs, Switzerland), bovine serum albumin (BSA, #9048-46-8, VWR Chemicals, Dietikon, Switzerland), and mouse non-swiss albino serum (#IMSNSASER100M, Innovative Research, Novi, MI, USA) were commercially purchased.

The human recombinant proteins used in this study; monomeric biotherapeutic candidate (26 kDa, called “Biologic” throughout the study); corresponding endogenous, dimeric binding partner of the Biologic (38 kDa, called “antigen” throughout the study); and both monoclonal anti-Biologic antibodies (mABA1 and mABA2 with molecular weights of ~140 kDa and rabbit IgG frameworks) were internally produced showing ≤3% aggregation after purification.

Polyclonal anti-biologic antibodies (pABAs) were generated by hyper-immunizing rabbits with the biologic protein according to standard protocols. Sera were collected at multiple time-points. Polyclonal ABAs were purified from pooled sera by affinity chromatography on a column against immobilized biologic protein.

### 2.2. Sample Preparation

Stressed biologic material was produced by spiking the biologic material in PBS (45× dilution). Stressed sample and control containing plain PBS were sealed and stored in a climate chamber (Infors HT, Basel, Switzerland) at 37 °C for 4 weeks.

For the subsequent analyses, the stressed biologic candidate was further diluted in PBS and in mouse serum, respectively.

### 2.3. Preparation of Antigen-Drug and Immune Complexes (ICs)

Complexes between antigen, biologic protein, and monoclonal and polyclonal anti-biologic antibodies were formed in PBS and in mouse serum (0.72% final concentration), respectively, according to molar ratios indicated in the result section. For assessment of complexes by SEC(-MALS) and SV-AUC, respectively, the concentration of individual components was 150 µg/mL or higher to accommodate the low sensitivity of the UV detectors. To allow the formation of the binding equilibrium, the individual complex mixtures were incubated at room temperature for 1 h prior to the performance of analyses.

### 2.4. (Differential) Size Exclusion Chromatography (dSEC)

All size exclusion chromatography experiments were performed on Waters Acquity Classic UPLC, equipped with a multi-wavelength detector. Protein species separation by an isocratic elution was achieved using a flow rate of 0.4 mL/min on a BEH SEC column (200 Å, 1.7 µm, 4.6 mm × 150 mm, Waters, Baden, Switzerland) maintained at 40 °C. The mobile phase was 50 mM sodium phosphate, 400 mM sodium perchlorate, and pH6.0. The detection of signals was performed at 280 nm.

The obtained chromatograms of individual protein components and complexes in PBS matrix were evaluated directly. For complexes incubated in a matrix containing mouse serum, a subtraction procedure was introduced. Hereby, chromatograms of mouse serum control samples (addition of PBS instead of protein components) were subtracted from the chromatograms of samples containing formed complexes in mouse serum matrix prior to the data interpretation. Data collection, evaluation, integration, and processing by subtraction were performed with Chromeleon 7.3 (Thermo Scientific, Reinach, Switzerland) software.

### 2.5. Size Exclusion Chromatography with Multi Angle Light Scattering Assessment (SEC-MALS)

Size exclusion chromatography with multi-angle light scattering assessment was performed for samples in PBS matrix background, but not in serum containing background, due to the strong interference of serum components in light scattering.

All SEC-MALS experiments were performed using the SEC procedure described above with the single deviation of using at least 10 µg sample to obtain a stable and reproducible light scattering signal for low-abundance species. MALS analysis was performed using MALS (microDawnTREOS, Wyatt, Dembach, Germany) and RI (Optilab UT-rEX, Wyatt, Dembach, Germany) detectors. The MALS instrument was calibrated with 99.8% anhydrous toluene (Merck, Darmstadt, Germany). The normalization was performed with a BSA standard. The same standard was used to perform the inter-detector delay correction. The refractive index (RI) data was used as the concentration source and a refractive index increment (dn/dc) value of 0.185 mL/g was applied. MALS data acquisition and evaluation were performed with Astra 8 software (Wyatt, Dembach, Germany).

### 2.6. Sedimentation Velocity-Analytical Ultracentrifugation (SV-AUC)

Analytical ultracentrifugation was performed with an Optima AUC analytical ultracentrifuge (Beckman, Krefeld, Germany) supplied with absorbance and interference optics. Four-hundred-and-eighty microliters of each sample and 480 µL PBS as reference (for interference measurements) were loaded into assembled cells with sapphire windows and 12-mm path length charcoal-filled epon double sector centerpieces. The samples were analyzed at 50,000 rpm in an eight-hole Beckmann-Coulter AN50-Ti rotor. Sedimentation was monitored at 280 nm or 300 nm and continuously scanned with a radial resolution of 10 microns taking one replicate per time point. Data analysis was carried out using the software Ultrascan 4 (Borries Demeler, University of Lethbridge, Lethbridge, AB, Canada). Intensity data were subjected to 2DSA analysis with meniscus and iterative fitting followed by PCSA with 100 Monte-Carlo iterations. All calculations were done in-house on a Ryzen128-1019 Gigabyte TRX40 AORUS PRO with an AMD Ryzen Threadripper 3990X 2.9 GHz 64 Core/128 Threads CPU.

### 2.7. Mass Photometry

Mass photometry analysis and data evaluation procedures are described in detail elsewhere [19]. In short: The sample chambers made of coverslips (high precision 24 × 50 mm No. 1.5 coverslips, CG15KH, Thorlabs, Dortmund, Germany) (No. 1.5, 24 × 24 mm^2^, VWR, Dietikon, Switzerland) and gasket wells (reusable CultureWell™ gaskets, wells 50, diam. × depth 3 mm × 1 mm CW-50R-1.0, Grace Bio-labs, Bend, OR, USA) were prepared according to the standard procedure and used for a single analysis. All measurements were performed on OneMP mass photometer (Refeyn Ltd., Oxford, UK). Data acquisition was performed using AcquireMP (v2.4.2, Refeyn Ltd., Oxford, UK). Data acquisition time was 60 s and sampling frequency was 10 Hz. Images were time averaged 5-fold and pixel binned 4 × 4 before saving. BSA and thyroglobulin were used as molecular weight calibration molecules, which were analyzed/measured at the beginning and the end of each experimental sequence (calibration using both landing density values and linear fit of the contrast-to-mass data).

Protein stock solutions and buffers were allowed to accommodate to the room temperature for at least 60 min before use in experiments. First, freshly prepared PBS was added to the chamber to identify and secure focal position for the entire measurement with an autofocus system based on total internal reflection. Then, freshly prepared sample (~200 to 1000 nM total concentration) was added for a total volume of 20 µL and the acquisition started immediately. Each sample was measured at least three times independently (*n* ≥ 3).

All MP images were processed and analyzed using DiscoverMP (v2.4.2, Refeyn Ltd., Oxford, UK). MP contrast distributions were plotted as Kernel Density Estimates (KDE) with a bin width set to 0.0002. For all plots, the KDE contrast bandwidth was set to: 0.00 to −0.06 contrast units. The integration and quantification of identified species on molecular mass distribution histograms was performed with the DiscoverMP software (v2.4.2, Refeyn Ltd., Oxford, UK).

### 2.8. Dynamic Light Scattering (DLS)

DLS analysis of proteins and immune complexes was performed using DynaPro PlateReader II (Wyatt, Dembach, Germany). Prior to the analysis, 30 µL of each sample was placed in triplicates on a 384-well microtiter plate (Corning #3540, no lid). The plate was centrifuged for 2 min at 2000× *g* at 20 °C (Heraeus Multifuge X3R, Thermo Scientific, Reinach, Switzerland) and placed in the DLS instrument. The Software Dynamics (Wyatt, Dembach, Germany) version 7.10.1.21 was used to schedule and automate the sample measurement using 15 acquisitions per single assessment with a duration of 5 s each at a constant temperature of 20 °C. Data analysis was performed using Dynamics (Wyatt, Dembach, Germany) to visualize the regularization graphs. Autocorrelation analysis was performed to assess the hydrodynamic radii of detected protein species.

## 3. Results

### 3.1. Setup of the Analytical Study

For the conducted study, we chose an immunoglobulin domain-based, monomeric protein, which contains engineered inserts in the amino acid sequence. This therapeutic protein construct, called as “biologic” throughout the study, was selected having in mind the generation of an in-vitro IC assessment tool-box applicable to biotherapeutics of any composition and accounting for protein-engineering-based modifications. Its endogenous, pharmacologic target is a dimeric, soluble protein, which is named “antigen” throughout the study. As surrogates for real, patient-derived ADAs, antibodies were generated, which specifically recognize the biologic. To make a differentiation to patient-derived ADAs, these antibodies are named “anti-biologic antibodies” (ABAs) throughout the study. Besides polyclonal ABAs (pABAs), two monoclonal ABAs (mABAs) were generated, which recognize distinct epitopes of the biologic and do not hinder each other from co-binding.

The interaction and complex formation studies were performed starting with the most simple protein-interaction combinations, followed by the addition of further factors to the system. The sequential analysis allowed for insights into the mechanistic aspects of the chosen IC system.

### 3.2. Assessment of Basic Interactions Relevant for Initiation of Immune Complex Formation

To obtain a general understanding about most basic complexes, which can be formed with only two of the individual protein components, equimolar mixtures were generated and analyzed. PBS was used in the mixtures as a buffering system to mimic physiological conditions. This simplified system allows to focus on the main immune complex components under optimal analytical method performance with clear data interpretation, without the challenges related to the use of biofluids as matrix.

**Figure 1:**
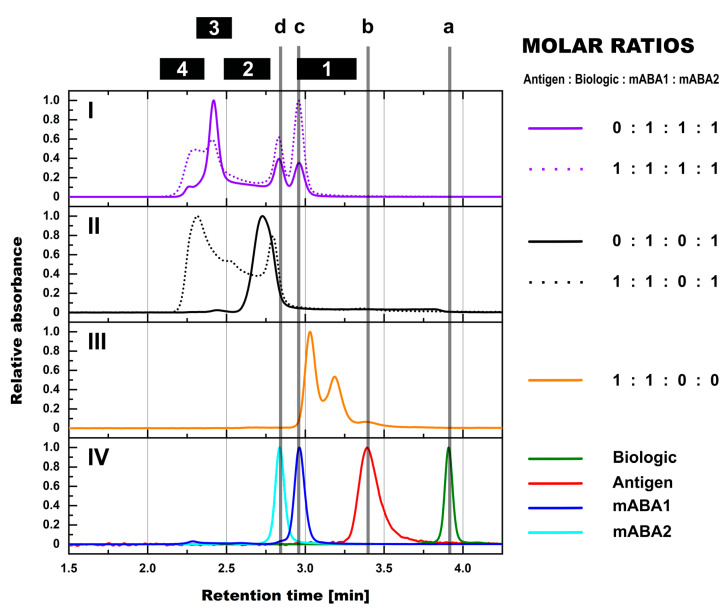
SEC-UV assessment of complex formation between biologic, antigen, and mABAs in PBS matrix. SEC profiles from equimolar mixtures are shown for biologic:mABA1:mABA2 ± antigen on panel (**I**), for biologic:mABA1 ± antigen on panel (**II**), and for antigen:biologic on panel (**III**). Panel (**IV**) shows controls, i.e., biologic in green, antigen in red, mABA1 in blue, and mABA2 in cyan. Marker: (a) free biologic, (b) free antigen, (c) free mABA1, (d) free mABA2, (1) antigen:biologic complexes, (2) immune complexes with a single ABA incorporated, (3) immune complexes with two incorporated ABAs, (4) immune complexes with more than two incorporated ABAs.

On the chosen SEC platform, the individual protein components can be readily distinguished by retention time (Figure 1). The MALS detectors were used to confirm the monodispersity of the individual protein components and to obtain the molecular weights (M_W_) for the formed complexes. For example, a relatively broad SEC peak was observed for the antigen material. Detection of monodispersity and a single molecular weight for the peak detected by MALS (data not shown) either suggests reversible self-association or unspecific interaction of the antigen with the column. Such interactions with the column are common for proteins with extended glycosylation [20], which is the case for the present antigen.

An equimolar mixture between the Biologic and the antigen formed two distinct complex species. Due to the dimeric character of the antigen, i.e., having two binding sites for the biologic, the main complex was composed of one antigen molecule and two biologic molecules. The presence of the additional 1:1 complex is related to the equimolar character of the mixture, where the biologic becomes the limiting factor.

The combination of the biologic with a mABA2 results in a single broad and rather distorted peak on SEC. Light scattering detected M_W_’s ranging from 140 to 200 kDa within the peak, indicating co-elution of free mABA2 with 1:1 and 1:2 mABA:biologic complexes. No species of higher M_W_’s were detected.

Mixtures containing the biologic and both mABAs formed complexes of even higher molecular weight (Figure 1, panel (I)), besides the previously observed complexes enclosing a single mABA. A clearly defined, main species with a measured M_W_ ranging from 300 to 350 kDa was observed by SEC-MALS. It can be attributed to complexes comprising two mABAs and up to three biologic molecules. Right after the void volume of the column, another distinct peak was detected (retention time: 2.2 to 2.7 min). Assessment by MALS classified this peak as a mixture of different species with M_W_’s ranging from 500 kDa up to 1–1.2 MDa. Lacking the resolution power in SEC, this peak could only be declared as a sum of “large” ICs containing three and more mABAs.

**Figure 2:**
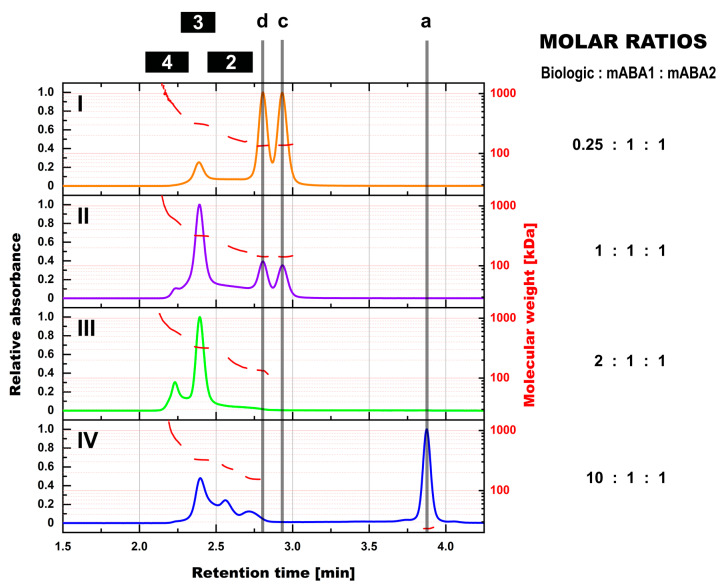
SEC-MALS assessment of formed immune complexes in PBS and the impact of the molar ratio between biologic and mABAs. Normalized SEC-UV profiles are shown for biologic:mABA1:mABA2 compositions of the following molar ratios—0.25:1:1 on panel (**I**), 1:1:1 on panel (**II**), 2:1:1 on panel (**III**), and 10:1:1 on panel (**IV**). Molecular weight profiles of detected immune complex species from MALS analyses are shown by scatter points in red. Marker: (a) free Biologic, (c) free mABA1, (d) free mABA2, (2) immune complexes with a single ABA incorporated, (3) immune complexes with two incorporated ABAs, (4) immune complexes with more than two incorporated ABAs.

Hereby, the ratio between the biologic and both mABAs is important for the equilibrium among the individual IC species (Figure 2). For any given combination, the IC species with two mABAs incorporated remained dominant. This was even the case in the 2:1:1 biologic:mABA1:mABA2 combination with all components being part of ICs (absence of free components). The highest amount of the non-dominant IC species with more than two incorporated mABAs was observed for the 2:1:1 combination. The level steadily declined when deviating from this component ratio. ICs with the incorporation of a single mABA were primarily present in combinations with mABAs being the limiting factor.

The combination of antigen, biologic and mABA(s) led to the formation of high M_W_ ICs (Figure 1, panels (I + II)). Hereby, two observations are of importance. Firstly, in comparison to biologic:mABA compositions, the addition of the dimeric antigen led to the (a) creation of new, high M_W_ ICs in the presence of a single mABA, and a (b) shift of the IC species equilibrium to high M_W_ ICs in the presence of both mABAs. These results show the impact of dimeric antigens in extending the cross-linking in ICs, forming ICs of upper M_W_’s and sizes. Secondly, competition occurred between the antigen and the mABAs for the binding to the biologic, which is observable as an increased amount of free mABAs not incorporated in the ICs (Figure 1, panel (I)).

An analysis of the above-mentioned basic components and their combinations forming ICs by SV-AUC showed similar results compared to SEC including the impact of the antigen to shift the equilibrium between the IC species to higher M_W_ values (Figure S1). With the used SV-AUC settings, focusing on immune complexes, low resolution power was observed for complexes of lower molecular weight. For example, the 1:1 and 1:2 antigen:biologic complexes could be detected by SV-AUC (Figure S1) but not resolved and therefore not reliably quantified. On the other hand, SV-AUC was superior to SEC-MALS in resolving IC species of higher molecular weight. SEC lacking the resolution power to discriminate IC species with more than two mABAs was outperformed by SV-AUC, which was able to resolve ICs containing 3, 4, and ≥5 mABAs, respectively. For the biologic:mADA1:mADA2 ± antigen compositions, clear discrimination of species with M_W_’s up to 3 MDa was observed with SV-AUC (Figure S1). Additionally, species at the end of the chosen S-range (50 S) were detected for the biologic:mADA1:mADA2 ± antigen compositions, indicating the formation of bigger aggregates and particles. This was confirmed by a cloudy appearance of those samples prior to the start of the runs.

DLS analysis of the above-mentioned compositions in PBS background was performed with commercial equipment, which is commonly used in the pharmaceutical industry. It was possible to discriminate between species representing single components and bigger complexes (Figure S2). Unfortunately, discrimination between the main IC species, as observed with SEC and SV-AUC for the same samples, was not possible. Due to the low-resolution power of DLS, low robustness of multimodal distribution analysis, and the mainly qualitative character of the results, analysis of samples containing ICs by DLS was not further continued.

### 3.3. Assessment of Immune Complexes in Presence of Polyclonal Anti-Biologic Antibodies

Subsequently, the basic immune complex platform was expanded, exchanging the two monoclonal ABAs by rabbit polyclonal anti-biologic antibodies (pABAs). The surrogate polyclonal antibodies are expected to mimic epitope-diverse anti-drug-antibody (ADA) populations in patients, providing a more realistic and meaningful insight into immune complex formation.

**Figure 3:**
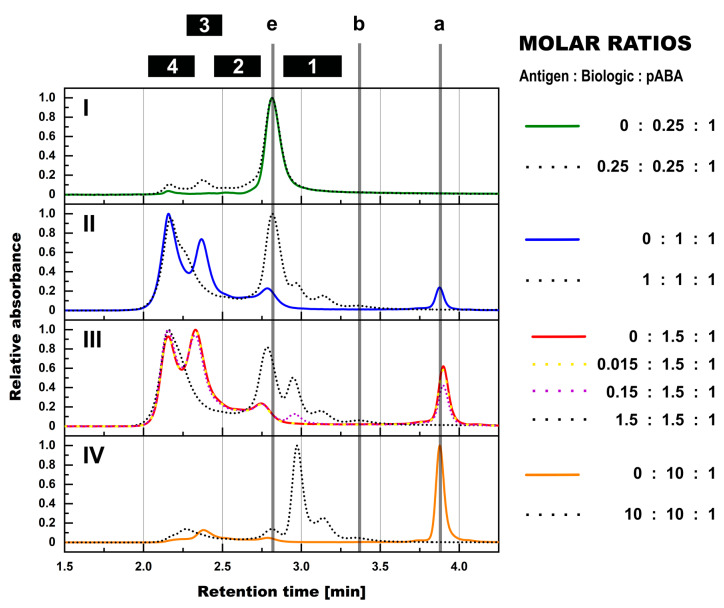
SEC assessment of formed immune complexes in PBS in presence and absence of antigen and the impact of molar ratios between biologic and pABA. Normalized SEC-UV profiles are shown for biologic:pABA compositions of the following molar ratios in absence (full line) and in presence of antigen (dotted lines)—0.25:1 on panel (**I**), 1:1 on panel (**II**), 1.5:1 on panel (**III**), and 10:1 on panel (**IV**). Presence of antigen in equimolar ratio to biologic and according to figure legend for the 1.5:1 biologic:pABA ratio. Marker: (a) free biologic, (b) free antigen, (e) free pABA, (1) antigen:biologic complexes, (2) immune complexes with a single ABA incorporated, (3) immune complexes with two incorporated ABAs, (4) immune complexes with more than two incorporated ABAs.

From the qualitative perspective, SEC-MALS experiments using pABAs instead of mABAs in combination with the biologic ± antigen showed a formation of IC species with equivalent M_W_’s (Figure 3). ICs containing one, two, and >two pABAs could be clearly distinguished.

Compositions using pABAs instead of mABAs clearly differed in terms of observed shift in relative abundance of ICs to species of higher M_W_’s. The immune complex species, which contained >2 ABAs, was the dominant form for the biologic:pABA composition at the equimolar ratio (Figure 3, panel (III)). The IC species containing two ABAs, which was dominating in the presence of mABAs at the same molar ratios (Figure 2, panel (II)), showed decreased abundance. It can be speculated that the polyclonal ABAs contained antibody sub-populations, which recognized a higher number of different epitopes on the biologic compared to the two distinct epitopes recognized by the mABAs. As a consequence, a different stoichiometry between the IC components and more flexible spatial IC architectures was becoming possible, leading to the stabilization of ICs of very high M_W_’s. Another observation using pABAs instead of mABAs was the higher content of free anti-biologic antibodies and the corresponding lower content of ICs. Again, this can be attributed to the higher variation within the pABA population. Competition of pABAs for binding to the same biologic molecule is expected for pABA sub-populations with overlapping recognition of epitopes. Additionally, in comparison to the two high affinity mABAs, the pABA population contained binders with a wide range of binding affinities. Especially the low affinity, transient binders might not be captured by SEC.

The addition of the antigen to the compositions containing biologic and pABAs led to a shift of ICs to larger species. Besides, the dominant IC species with more than two pABAs, a new, slightly smaller IC species was observed. Its retention time (~2.26 min, Figure 3) ranged between the ICs containing two and >two pABAs. Due to the partial co-elution with ICs with >two pABAs, the species could not be quantified, nor directly attributed, due to the absence of reliable M_W_ measurement by MALS. Most probable compositions would be antigen:biologic complexes, which are cross-linked by two to three pABAs. This speculation is supported by an almost complete disappearance of the 2–3× biologic:2× pABAs species, which was observed in the absence of the antigen as one of the main IC species.

As seen before for mABA:biologic compositions, extensive competition for binding to overlapping epitopes between specific pABA species and the antigen was observed via a high content of free pABA in the profiles (Figure 3). This finding is in line with reports describing the CDR regions of therapeutic antibodies and similar formats being the most immunogenic parts of the biological drugs [2,21].

The variance of the molar ratios between all three components, i.e., antigen, biologic and ADA, led to numerous implications for the IC formation, but was following common rules. In the molar ratios with pABA being the dominant component over the biologic (Figure 3, panel (I)), all of the biologic molecules were incorporated into ICs. The addition of the antigen to such a composition led to a complete incorporation of the antigen in the ICs. In excess of biologic, i.e., free biologic content observed in SEC profiles (Figure 3, panels (I–III)), the addition of an antigen at non-limiting levels led to two effects: free biologic molecules were incorporated into antigen:biologic complexes. Moreover, the antigen molecules were incorporated into ICs in a competitive manner, i.e., displacement of pABA molecules from ICs and, herewith, an increase in levels of free pABA. The impact of antigen being the limiting factor was assessed in the 1.5:1.5:1 antigen:biologic:pABA molar ratio (Figure 3, panel (II)), reducing the amount of antigen to 10 and 1% of the initial amount, respectively. In comparison to the composition lacking the antigen, the addition of the “10%-antigen amount” led to the consumption of the antigen forming the 1:2 antigen:biologic complex species at the expense of free biologic, but without any effect on the present biologic:pABA ICs. Reducing the amount of antigen to 1% led to a SEC profile, which could not be distinguished from the 1.5:1 biologic:pABA SEC profile.

**Figure 4:**
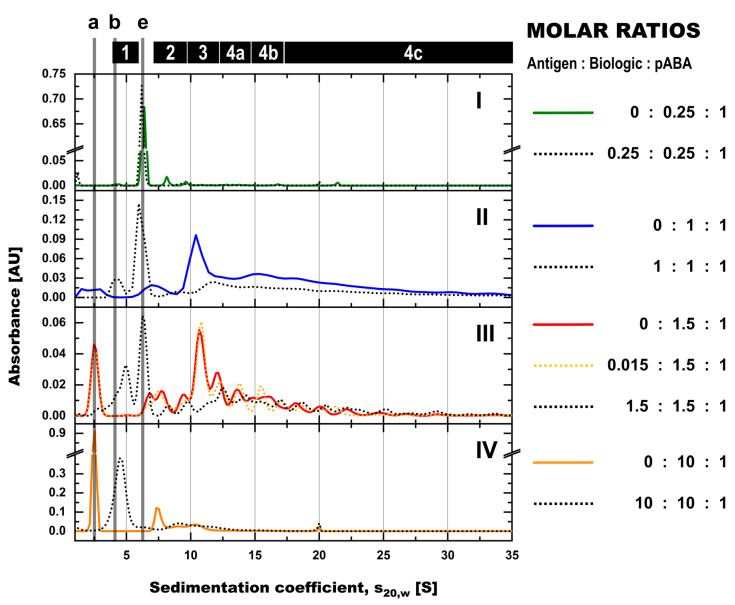
SV-AUC assessment of formed immune complexes in PBS in presence and absence of antigen and the impact of molar ratios between biologic and pABA. SV-AUC profiles are shown for biologic:pABA compositions of the following molar ratios in absence (full line) and in presence of antigen (dotted lines)—0.25:1 on panel (**I**), 1:1 on panel (**II**), 1.5:1 on panel (**III**), and 10:1 on panel (**IV**). Presence of antigen in equimolar ratio to biologic and according to figure legend for the 1.5:1 biologic:pABA ratio. Marker: (a) free biologic, (b) free antigen, (e) free pABA, (1) antigen:biologic complexes, (2) immune complexes with a single ABA incorporated, (3) immune complexes with two incorporated ABAs, (4a) immune complexes with three incorporated ABAs, (4b) immune complexes with four incorporated ABAs, (4c) immune complexes with more than four incorporated ABAs incorporated.

The analysis of biologic:pABA compositions ± antigen by SV-AUC confirmed SEC findings and provided further insights, due to the higher resolution in the M_W_ range above 500 kDa. For biologic:pABA compositions with deviation from the equimolar situation, i.e., 0.25:1 and 10:1 (Figure 4, panels (I + IV)), SV-AUC confirmed that the formed ICs were mainly small, containing one to two ABA molecules, irrespective of the presence or absence of the antigen. Whereas, for the compositions at or near to the equimolar biologic:pABA compositions, i.e., 1:1 and 1.5:1 (Figure 4, panels II + III), high M_W_ complexes ranging up to 35 S (corresponding to ~2.3 MDa) could be detected. The ICs containing >2 pABAs could be differentiated, but seemed to be less well defined in comparison to the ICs containing one or two ABAs. Similar to the results observed by SEC, the addition of the antigen to the biologic:pABA compositions led (a) to the consumption of free biologic forming antigen:biologic complexes and (b) to competition between antigen and pABA for the biologic that is incorporated in ICs. Specifically, reduction of the ICs containing one to two pABAs and an increasing amount of free pABAs were observed. The abundance of ICs with >2 pABAs was only marginally affected by the added antigen.

### 3.4. Assessment of Immune Complexes in Presence of Stressed Biologic Material

Deteriorated biologics material is known to have the potential to induce adverse events in patients, which is not observed with intact drug molecules [22]. To dissect whether deteriorated biologic would have an impact on IC formation, thermally stressed biologic was produced to mimic natural protein degradation, which might happen during extended shelf-life or during circulation in the body at 37 °C. IC formation from combinations of stressed material and pABA in the presence and absence of antigen was compared to IC profiles using non-stressed biologic.

**Figure 5:**
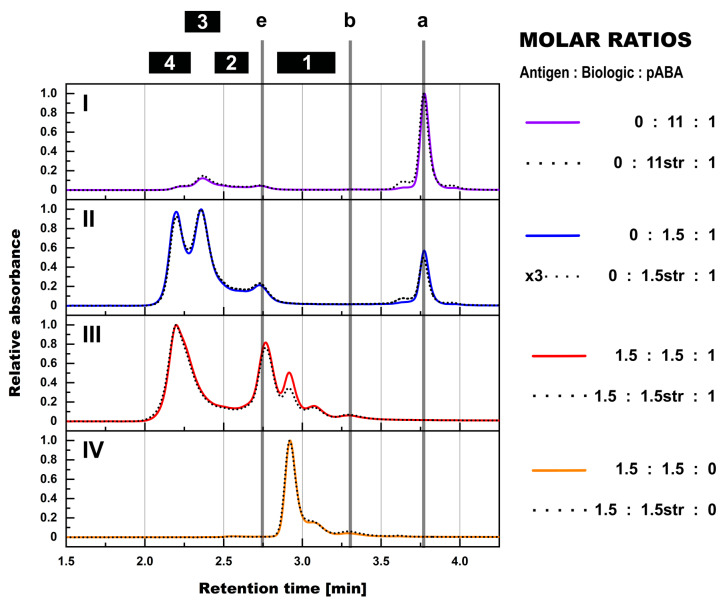
SEC assessment: impact of biologic material degradation on formation of immune complexes in PBS matrix. Normalized SEC-UV profiles are shown for antigen:biologic:pABA compositions of the following molar ratios—0:11:1 on panel (**I**), 0:1.5:1 on panel (**II**), 1.5:1.5:1 on panel (**III**), and 1.5:1.5:0 on panel (**IV**), whereby full lines represent compositions using non-stressed biologic material and dotted lines represent compositions using stressed material (performed in triplicate for the 0:1.5:1 composition). Marker: (a) free biologic, (b) free antigen, (e) free pABA, (1) antigen:biologic complexes, (2) immune complexes with a single ABA incorporated, (3) immune complexes with two incorporated ABAs, (4) immune complexes with more than two incorporated ABAs.

The impact of applied stress can be observed in SEC profiles of free biologic components for the stressed material (Figure 5, panel (I)). A minor species with an apparent, higher M_W_ running as pre-shoulder at a retention time of ~3.6 min was visible. SEC analysis with stressed material did not show an impact on the formation of antigen:biologic complexes (Figure 5, panel (IV)). Similarly, only a marginal impact on IC formation was observed using stressed material in combinations with biologic and pABA (Figure 5, panels (I–III)). The minor differences in profiles with and without stress are of relevance considering the very high precision of the method and the low variability, given the perfectly overlapping profiles of stressed material compositions prepared and analyzed in triplicates (Figure 5, panel (III)). Surprisingly, a more prominent impact of stressed material was observed when combining all three components, i.e., antigen, biologic, and pABA (Figure 5, panel (III)). A lower relative abundance was observed for the 1× antigen:2× Biologic complex, but not for any other species. One could speculate that stress-related modifications of the biologic have a modulating, but not an abrogating impact on the dissociation constant of the biologic for binding to its antigen.

**Figure 6:**
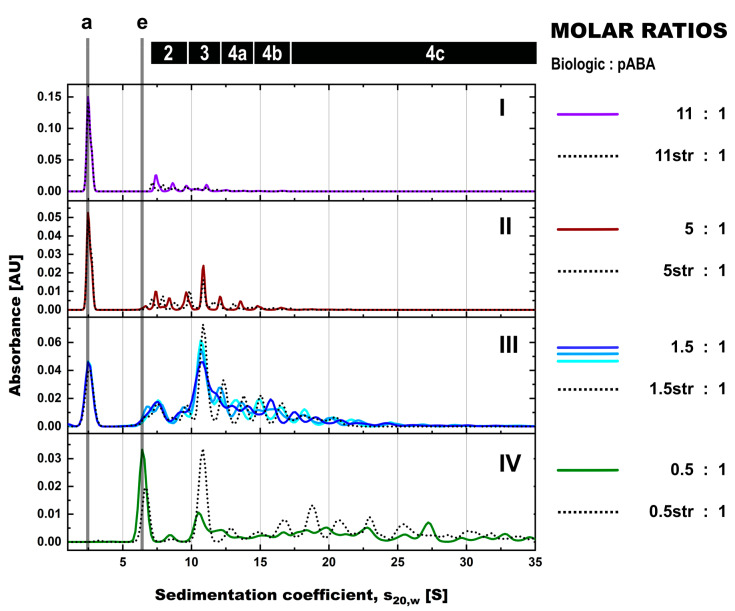
SV-AUC assessment: impact of biologic material degradation on formation of immune complexes in PBS matrix. SV-AUC profiles are shown for biologic:pABA compositions of the following molar ratios—11:1 on panel (**I**), 5:1 on panel (**II**), 1.5:1 on panel (**III**), and 0.5:1 on panel (**IV**), whereby full lines represent compositions using non-stressed biologic material (performed in triplicate for the 1.5:1 composition) and dotted lines represent compositions using stressed material. Marker: (a) free biologic, (e) free pABA, (2) immune complexes with a single ABA incorporated, (3) immune complexes with two incorporated ABAs, (4a) immune complexes with three incorporated ABAs, (4b) immune complexes with four incorporated ABAs, (4c) immune complexes with more than four incorporated ABAs.

Analysis of ICs using stressed vs. non-stressed biologic by SV-AUC showed only minor differences for individual biologic:pABA compositions, when pABA was the limiting factor (Figure 6, panels (I-III)). More substantial differences were observed for the ratio with the biologic being the limiting factor (Figure 6, panel (IV)). Higher levels of ICs containing two and more pABAs were observed in the presence of stressed material. The most pronounced change was the increase in the IC species containing 2 ABA molecules in the presence of stressed material, which was not observed for other tested molar ratios between biologic and pABA. Hereby, it is critical to not overestimate the differences observed with stressed vs. non-stressed biologic, when looking at a continuum of species with SV-AUC. The non-stressed biologic:pABA combination 1.5:1 was prepared and analyzed in triplicate (Figure 6, panel (III)). The free components and ICs containing up to 2 pABAs are quite reproducible with minor deviations in the shape and resolution of the peaks. Immune complex species containing more than 2 pABAs show high variation in shape and the S value of the detected species. Such variability is indicative for less defined ICs, which probably originate from random association of the components and not from geometrically stable structures.

### 3.5. Assessment of Immune Complexes at Low and Physiological Concentrations

Intravenously delivered biotherapeutics, even highly dosed and concentrated, have plasma concentrations in the upper nanomolar range [23]. The concentrations of newer biologic modalities like ADCs and bispecifics are significantly lower. The abundance of corresponding pharmacologic targets in the body is in most cases lower than the administered biologics. The concentration of drug-specific ADAs is dynamic and depends on many factors, but even in patients with present post-administration adverse events, ADA levels have been shown not to exceed the upper nanomolar range [23].

In-vitro analysis of immune complexes by SEC and SV-AUC without signal amplification procedures, e.g., utilization of fluorescence probes, does not allow for the analysis of protein complexes at low nanomolar concentrations due to sensitivity limitations of UV absorption and interference detection. Instead, a novel analytical tool, mass photometry (MP), was employed to gain insight into relevant aspects of immune complex formation at physiological concentrations.

The biologic used in this study had a K_D_ in the low picomolar range for the corresponding antigen. The available pABAs containing multiple sub-populations, although not experimentally confirmed, are expected to have a broad range of affinities. Experiments performed with our model system at this concentration range might be strongly influenced by the K_D_ of pABAs, which were lying in the same range.

MP has the best performance range in the single-digit to low three-digit nanomolar range [19] and is therefore applicable for the assessment of IC formation at physiological concentrations. Mass photometry monitors reflectivity changes that a biomolecule provokes, replacing water on a glass surface. Hereby, the ratiometric contrast of the detected landing events is directly proportional to the size of the detected molecules.

The molar ratio of 1:1 between biologic and pABA was selected to assess the impact of changing total protein concentration on the formation of immune complexes, specifically focusing on relative abundancies of individual IC species. Considering the content of both components, a concentration range between 12.5 and 102 nM was assessed (Figure S3). From the qualitative perspective, the analysis was showing the detection of distinct species, which corresponded in their M_W_ to the expected ICs. Single events up to 1.2 MDa were detected. The separation of individual IC species was sufficient for quantification. Limitations regarding overlapping signals were observed, discriminating free ABAs and IC complexes containing a single antibody molecule. Another limitation was related to the M_W_ detection limit, which is ≥~40–60 kDa [24]. For our system this corresponds to the size of free biologic, the antigen, and partly antigen:biologic complexes. The experiments showed some signal in this M_W_ range (<100 kDa), but these varied significantly when re-analyzing the same samples. Therefore, only species above 100 kDa were taken into account for quantification. Due to limited precision of a single assessment, multiple analyses of the same biologic:pABA composition were performed and averaged for the quantification. Overlays of the individual analyses (Figure S4) revealed pronounced differences in the abundancies of the individual IC species. The observed challenge, which mass photometry shares with other single molecule analysis methods, is related to (a) technical limitations and (b) experimental systems with a high number of distinct species of varying abundancies. Taking into account the single-digit numbered relative abundance of high M_W_ IC species, the number of detected events was correspondingly low and therefore imprecise. Analysis at higher concentrations, aiming to improve precision, was not feasible, due to overlapping events of species with high abundance (Figure S3). Thus, the comparative analysis of the biologic:pABA composition 1:1, varying the total protein concentration by a factor of ~8, showed clear differences. It remains unclear whether these differences originate from the described technical limitations, the impact of affinity constants on formation of ICs, or from the combination of both.

**Figure 7:**
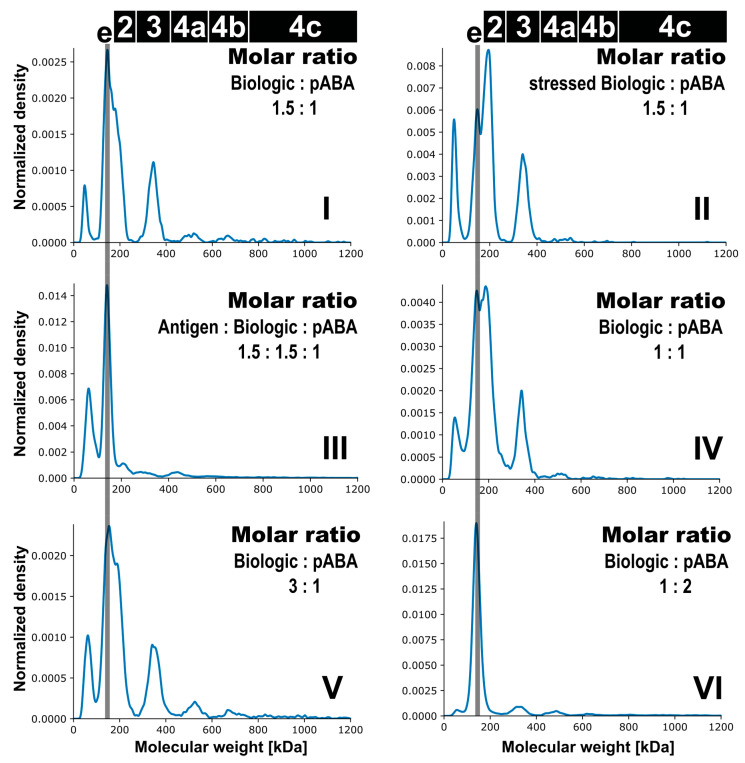
Analysis of immune complex formation at physiological concentrations. Mass photometry kernel density estimate profiles are shown for antigen:biologic:pABA compositions of the following molar ratios—0:1.5:1 on panel (**I**), 0:1.5 (stressed biologic):1 on panel (**II**), 1.5:1.5:1 on panel (**III**), 0:1:1 on panel (**IV**), 0:3:1 on panel (**V**), and 0:1:2 on panel (**VI**). Marker: (e) free pABA, (2) immune complexes with incorporation of a single ABA, (3) immune complexes with two incorporated ABAs, (4a) immune complexes with three incorporated ABAs, (4b) immune complexes with four incorporated ABAs, (4c) immune complexes with more than four incorporated ABAs.

Next, the impacts of (a) stressed biologic, (b) presence of the antigen, and (c) difference in molar ratios between the biologic and pABA on formation of ICs were assessed (Figure 7). To allow comparability assessment between tested conditions, the concentration of the pABA component was fixed and the concentrations of all other components adapted to reach specific molar ratios. Similar to the results from SEC and SV-AUC experiments, limited impact of stressed biologic on the formation of ICs was observed for the biologic:pABA composition with a molar ratio of 1.5:1. The observed differences are related to quantities of the present species, mainly the smallest IC containing a single ABA. The addition of the antigen led to a decrease in amounts of ICs, with the reduction of ICs containing two ABAs being most pronounced. Although not quantified, a clear increase in the amount of species with M_W_ < 100 kDa was observed. Changing the molar ratio between Biologic and pABA to 3:1 had a low impact on the relative abundance of ICs (≥2 ABAs incorporated). Regarding the equimolar ratio between the biologic and pABA, the highest abundance was observed for ICs containing a single ABA. The change of the molar ratio, with the biologic becoming the limiting factor, i.e., 1:2, resulted in substantially decreased levels of any ICs. In contrast, at high protein concentrations, the 1:2 ratio translated into the highest relative abundance of formed high M_W_ ICs containing three and more ABAs (Figure 6). This result can only be partially explained by the previously indicated IC signal quenching by the dominant free pABA amount.

Cumulatively, it can be stated that the total protein concentration had a noteworthy impact on the formation of immune complexes. This was not observed on the qualitative level, since MP was detecting the same IC species as SEC and SV-AUC, the latter of which were performed at elevated protein concentrations. However, the relative abundances of ICs were substantially lower at physiological concentrations. Additionally, the distribution between the identified IC species was shifted predominantly to smaller ICs. These were mainly composed of one to two pABAs, followed by a minority of larger ICs species containing >2 pABAs.

### 3.6. Assessment of Immune Complexes in Serum Background

The above-described experiments were fully focused on intrinsic parameters of IC formation such as the concentration or the ratio of individual components. Studies have reported an impact of extrinsic parameters on the formation of ICs, e.g., observation of different IC structures formed in PBS and in serum background [25]. The main aim of the present study was to mimic in-vivo conditions as closely as possible by in-vitro experiments. More specifically, the goal was to obtain an in-vitro platform with the ability to predict characteristics of in-vivo immune complex formation at specific time-points post administration of the biological drug (steady state).

The analysis of ICs and corresponding free components in biological matrices such as serum poses several challenges. Aside from the in-vitro instability of serum [15], the main challenge is related to the specificity of analytical methods. Most of the quantitative analytical methods with separation character, aiming for the assessment of protein molecular weights and relative abundance of individual species, are relying on UV light absorption. In the case of proteins, this is connected to absorbance by peptide bonds and aromatic amino acids. Consequently, the detection by UV is unspecific to the proteins of interest. Serum components strongly interfere with UV-based analyses. The total protein concentrations of biofluids are high, reaching up to 80 mg/mL [26], with human serum albumin and antibodies being the most abundant species. Several approaches for method adaptations have been reported, bypassing the challenge of interference posed by serum components, but do not eliminate all of the limitations [27]. For example, the most promising of the approaches, i.e., protein fluorescence labeling, might have an impact on the physico-chemical properties of the proteins of interest and might modulate or in worst case impair binding to interaction partners.

**Figure 8:**
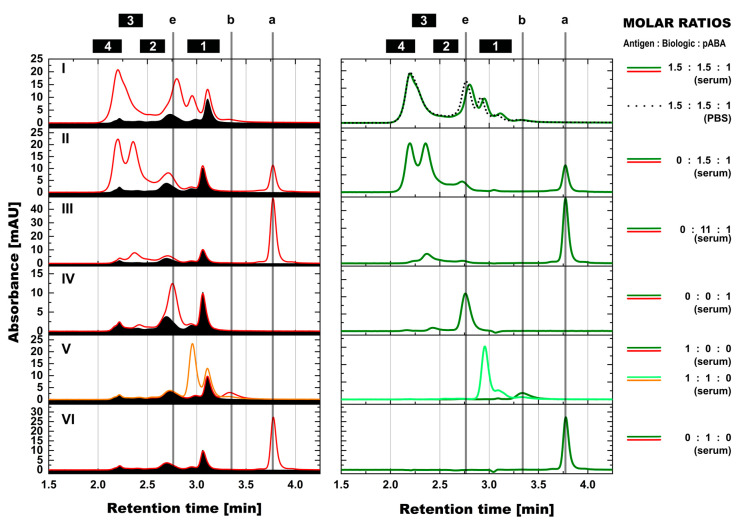
Assessment of immune complex formation in mouse serum background by SEC. SEC profiles of diluted mouse serum represented by black area under the curve and samples in diluted mouse serum by red lines (left panels), and profiles of differential representation of both after subtraction of serum matrix by green lines (right panels). Antigen:biologic:pABA compositions of the following molar ratios are shown—1.5:1.5:1 on panel (**I**), 0:1.5:1 on panel (**II**), 0:11:1 on panel (**III**), 0:0:1 on panel (**IV**), 1:0:0 and 1:1:0 on panel (**V**), and 0:1:0 on panel (**VI**). Hereby, full lines represent compositions analyzed in mouse serum and dotted lines represent composition analyzed in PBS. Marker: (a) free biologic, (b) free antigen, (e) free pABA, (1) antigen:biologic complexes, (2) immune complexes with a single ABA incorporated, (3) immune complexes with two incorporated ABAs, (4) immune complexes with more than two incorporated ABAs.

**Figure 9:**
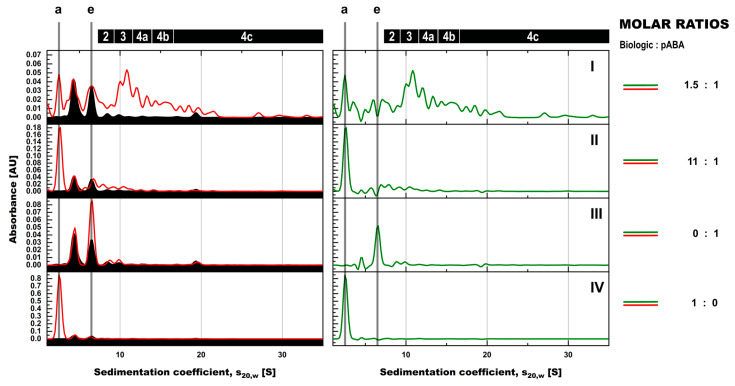
Assessment of immune complex formation in mouse serum background by SV-AUC. SV-AUC profiles of diluted mouse serum represented by black area under the curve and samples in diluted mouse serum by red lines (left panels), and profiles of differential representation of both after subtraction of serum matrix by green lines (right panels). Biologic:pABA compositions of the following molar ratios are shown—1.5:1 on panel (**I**), 11:1 on panel (**II**), pABA control on panel (**III**), and biologic control on panel (**IV**). Marker: (a) free biologic, (e) free pABA, (2) immune complexes with a single ABA incorporated, (3) immune complexes with two incorporated ABAs, (4a) immune complexes with three incorporated ABAs, (4b) immune complexes with four incorporated ABAs, (4c) immune complexes with more than four incorporated ABAs.

Aiming for unaffected interaction between components that are forming ICs, the direct analysis in serum containing all components was favored. To overcome the interference from serum components, mainly the oversaturation of the detectors, serum diluted with PBS was chosen as a surrogate of a biological matrix. To avoid data interpretation challenges from co-eluting/migrating serum components and to ensure correct quantification of the relevant protein species, serum blank controls were recorded in the same runs. The resulting signals were subtracted from profiles of samples containing antigen:biologic:pABA compositions in the same surrogate serum background (Figure 8 and Figure 9). Minor but negligible artefacts were observed after the applied subtraction procedure, which were related to sample preparation and technical performance differences. For the SEC runs (Figure 8), these artefacts were mainly related to the signal intensity of serum albumin (retention time of 3.1 min), only interfering with antigen:biologic complexes and not with any immune complex species. In the SV-AUC runs (Figure 9), besides the intensity-related artifacts (pipetting differences), also slight migration shifts related to non-ideality behavior of the solutions could be observed. Most prominent artefacts were related to the most abundant serum species with molecular weights <200 kDa (limited absorption of serum components of higher molecular weight at 280 nm). The resolution power of SV-AUC is limited in this “low” molecular weight range. Therefore, these artefacts do not impact on the focus of the SV-AUC assessment dramatically, namely detection and quantification of present immune complex species.

To get a full overview over the performance of analytical methods used in this study, it must be noted that several methods are incapable in the analysis of protein components in biofluids. MALS is not suitable to analyze protein complexes in serum background due to strong, interfering light scattering originating from high molecular weight components of the serum. Drastic analytical challenges are also encountered with mass photometry in the presence of serum. The concentration of the proteins of interest must be equal or higher compared to the total protein concentration of the serum background to get a suitable signal of proteins of interest for a qualitative assessment. Similar considerations are also true for DLS.

Both suitable methods for the assessment of ICs in serum, SEC and SV-AUC, showed agreement in the formation of IC species driven by the molar ratio between the components, i.e., antigen, biologic, and pABAs. The formed complex species and their quantities were comparable to the findings observed in PBS background. Top panel of Figure 8 shows almost identical, overlayed profiles for the molar ratio composition of 1.5:1.5:1 of antigen:biologic:pABA in PBS and in surrogate serum background. For the studied immune complex system, the serum background had only a limited impact, if any, on the formation of the immune complexes.

## 4. Discussion

Over the last decade, a tremendous increase in knowledge was obtained in the field of immunologic processes. Revealing the underlying mechanisms and cascades [2,16], it improved the understanding of immunogenicity and related adverse events in patients following the administration of biotherapeutics. Many protein biotherapeutics provoke the formation of anti-drug antibodies to some degree, while for only few of them, the formation of ADAs leads to severe side effects [16,28]. The activation of the adaptive immune system drives the formation of high-affinity ADAs by somatic hypermutation. Under specific conditions, the ADAs lead to the formation of ICs by the interaction with the biologic molecules. Elimination of the circulating ICs is governed by regular processes such as phagocytizing cells internalizing the ICs via Fcγ receptors or elimination of ICs via opsonization by the complement system.

Multiple studies have shown that the initiation and the severity of the downstream effects of immune complexes are dependent on their size, composition, and structural conformation [29,30]. In-vitro experiments indicate that phagocytosis by macrophages is only initiated by ICs containing ≥ 4 antibodies, whereas activation of the complement system by binding of C1q to the ICs requires at least six antibodies incorporated in the ICs [23]. When present in sufficient amounts and sizes, ICs can have effects on various cell types and induce their activation.

These examples suggest that a mechanistic understanding of the processes governing IC formation including a reliable methodology to characterize these complexes is a pressing need. The limited snapshot view on the contents of present ICs, free biotherapeutic and free ADA, that can be obtained from the analysis of patient-derived serum samples, is restricted by multiple factors: (1) the complexity and dynamic characteristics including clearing & sustaining of ICs and (2) the limited capabilities to analyze and characterize efficiently the individual variants in the relatively low amounts of present ICs compared to the abundance in other proteins present in plasma or serum.

In-vitro assessments are able to shed light on factors modulating IC formation. These factors are clearly not of generic applicability, but are dependent on the class of the biotherapeutic [14], and sometimes even on the specific biotherapeutic candidate. Therapeutic mAbs trigger mainly anti-idiotopic immune responses, with the ADAs being mainly directed against the CDR regions and not against the human framework of IgGs [21,31]. Non-mAb biotherapeutics can introduce additional complexity regarding IC formation, due to protein engineering, for example via point mutations or fusion of domains, which can lead to the formation of additional epitopes and result in additional ADA populations, providing opportunities for the formation of ICs of new geometries and sizes.

**Figure 10:**
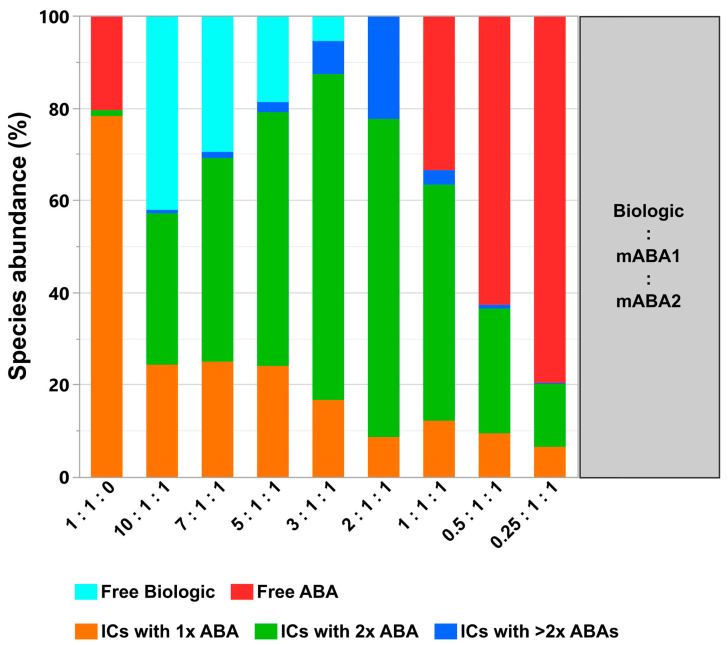
Quantification of formed immune complexes (ICs) between biologic and two monoclonal anti-biologic antibodies (mABAs). Quantitative SEC-MALS analysis read-out assessing relative levels of formed complexes and free components. Analyzed mixtures contain indicated molar ratios between biologic, mABA1, and mABA2 in PBS as matrix.

**Figure 11:**
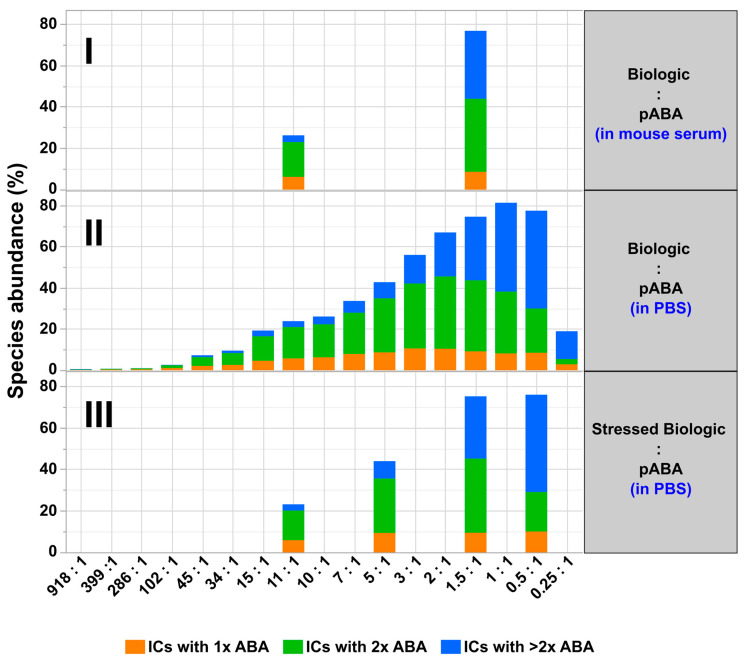
Quantitative SEC analysis read-out assessing relative levels of formed immune complexes between biologic and pABA. Analyzed mixtures are shown containing indicated molar ratios between biologic and pABA in diluted serum background on panel (**I**) and in PBS on panel (**II**). Quantified immune complex amounts using stressed biologic (4 weeks at 37 °C in PBS) are shown on panel (**III**). Detected amounts of free components are not represented in the graph.

**Figure 12:**
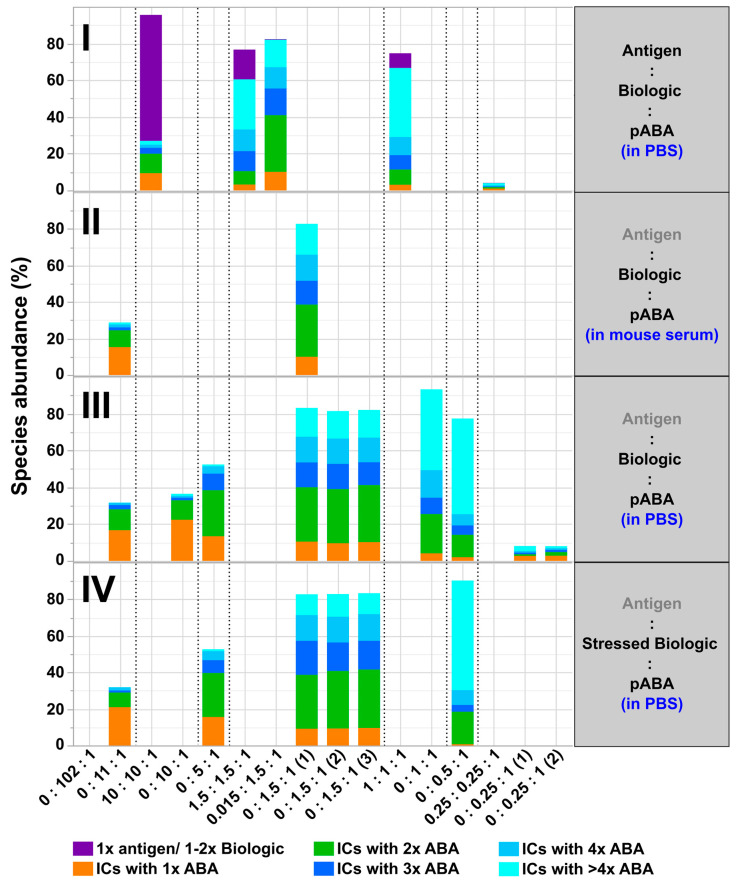
Quantitative SV-AUC analysis read-out assessing relative levels of formed immune complexes between antigen, biologic, and pABA. Analyzed mixtures are shown containing indicated molar ratios between biologic and pABA in the presence of the antigen in PBS background on panel (**I**), in diluted serum background on panel (**II**), in PBS on panel (**III**). Quantified immune complex amounts using stressed biologic (4 weeks at 37 °C in PBS) are shown on panel (**IV**). Detected amounts of free components are not represented in the graph.

**Figure 13:**
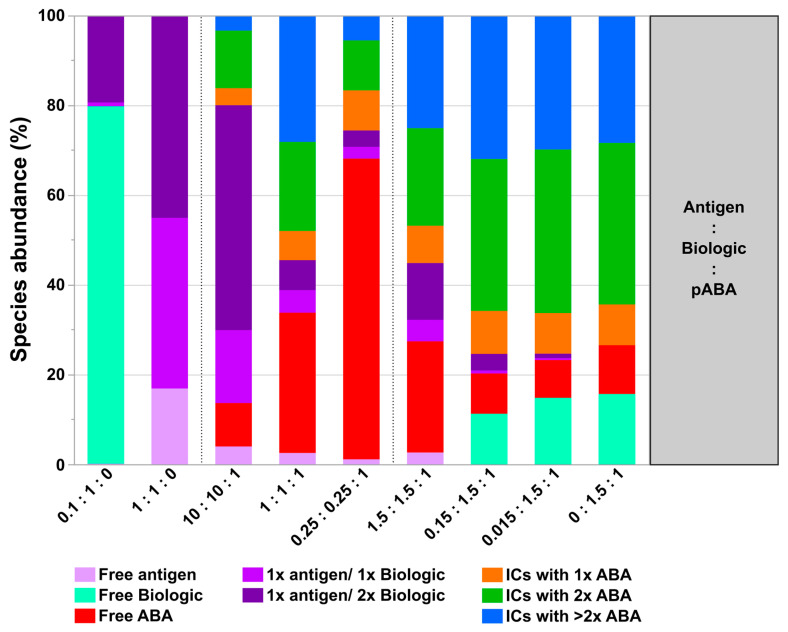
Quantitative SEC-MALS analysis read-out assessing relative levels of formed complexes between antigen, biologic and pABA in PBS background. Analyzed mixtures contain indicated molar ratios between the components.

All of the described aspects and increased immunogenicity risks with new biotherapeutic formats emphasize the need for early in-vitro IC formation & immunogenicity pre-assessments of biotherapeutic candidates by analytical tools described in this study. Our model system and the experimental design have demonstrated a complex interplay between factors cumulatively responsible for the formation, abundance, and sizes of ICs. The following important factors can be highlighted:

1. The most simple test concept between a monomeric biotherapeutic and anti-biotherapeutic mAbs highlights the necessity for having at least two anti-biotherapeutic antibodies, which recognize different and non-overlapping epitopes, to generate ICs larger than a 1:1 biotherapeutic:Ab complex. Alternatively, for multimeric biotherapeutics like therapeutic mAbs, an ADA against a single epitope on the therapeutic mAb is sufficient for the formation of high M_W_ ICs by cross-linking of multiple therapeutic Abs and multiple ADAs. As expected, for the monomeric biologic used in this study, in the presence of two mABAs, which recognize non-overlapping epitopes, the highest amount of ICs (=full conversion of single components) was observed for the equimolar 2:1:1 molar ratio (Figure 10). Under these conditions, also the amount of ICs with higher M_W_ was largest, but decreased dramatically with a molar excess of either the biologic or the mABAs.
2. The expansion of the simplistic model through the exchange of mABAs by surrogate pABAs, which have the potential to cover a broader epitope space, increases the number of options to generate new and more complex forms of ICs. In our hands, the highest levels of ICs were observed at or near to equimolarity between the biologic and the pABA (Figure 11 and Figure 12) in agreement with published data on other IC systems [23]. Our experiments indicate that the most stable complex is formed by cross-stabilization of IC species containing 2 ABAs, presumably avidity driven by all four Fab-arms being involved in the interaction with the biologic molecules. Surprisingly, a non-Gaussian distribution of IC content was observed measuring the abundance of ICs at various molar ratios between the biologic and pABA (Figure 11). The presence of ICs could be detected even at a ~1000-fold molar excess of the biologic over pABA. In contrast, the content of ICs decreased rapidly with molar excess of pABA over the biologic and could not be detected below an excess of pABA of 5-fold (Figure 11 and Figure 12). The ratio between the components was not only dictating the conversion rate of free components to ICs, but had a significant impact on the dynamic distribution between the individual IC species. The amount of smaller ICs, which contain one to two pABA molecules, was highest at a slight molar excess of the biologic (2:1 ratio), and were generally the dominating species at molar excess of the biologic. In contrast, the larger ICs (≥3 pABAs) became the dominant species with molar excess of pABA over the Biologic. The highest amount of larger ICs was observed for the 0.5:1 ratio at non-physiological concentrations.
3. Expanding the biologic:pABA system by the inclusion of a dimeric antigen provided insights into additional factors contributing to IC formation (Figure 12 and Figure 13). Our experiments demonstrate that the antigen was competing with sub-populations of pABA for free and complexed biologic molecules. In the presence of free biologic material (pABA being the limiting factor), antigen:biologic complexes were formed. In parallel, antigen molecules were getting incorporated into larger biologic:pABA complexes (ICs containing >2 pABA molecules). This led to an increased amount of these species at the expense of smaller ICs (containing ≤2 pABA molecules). Disintegration of the latter with subsequent freeing-up of non-incorporated pABAs was the observed result (Figure 3 and Figure 4). The preferable disintegration of smaller ICs by the antigen indicates an involvement of the biologic’s CDRs in the competitive binding between the antigen and the pABA species, which were involved in the smaller ICs, and/or conformational restrictions imposed by bound antigen. On the other hand, the incorporation of antigen molecules into larger ICs suggests mainly a non-idiotype-driven interaction of pABA species with the biologic in such complexes. Additionally, cross-linking of two biologic molecules by the dimeric antigen provided a two-fold increase in binding sites for pABAs (with exclusion of the CDR region), thereby allowing a higher degree of complexation and new conformational orientations between the components. These larger complexes must be of random conformation and not well structured. The absence of preferred and conformationally stable high M_W_ IC species was detected by strong variability in the amount of individual IC species containing three and more pABAs (Figure 6, triplicate analysis of same composition by SV-AUC). The significance of the antigen for the formation of ICs should not be overestimated. For most biotherapeutics with the aim to neutralize a pharmacological target, the concentration of the administered biologic in the patient is often orders of magnitude higher as compared to the concentration of the endogenous target. The in-vitro experiments show that a reduction in the molar concentration of the antigen in the antigen:biologic:pABA compositions from an equimolar ratio to 10% (=0.1:1 antigen:biologic ratio) strongly reduced the impact of the antigen on the IC composition (Figure 13). A reduction to 1% led to a negligible impact of the antigen on the IC formation.
4. In addition to the molar ratio, the absolute concentration of the components is a critical factor for IC formation. The analysis of ICs at low µmolar to high nanomolar (SEC and SV-AUC results) and at low nanomolar (mass photometry) concentrations showed a direct correlation between the concentration and the amount of formed ICs. Hereby, the reduction was relatively more pronounced for the amount of larger ICs (>2 pABAs incorporated) compared to the amount of smaller ICs. The analysis of ICs at low nanomolar concentrations is generally preferable due to a higher relevance for physiological conditions. A significant factor with the potential to impact IC formation and its composition is the K_D_ of ADA molecules to the corresponding target biotherapeutic. At least for low-affinity ADAs (early during the immune response), the K_D_ is reported to be in the medium to low nanomolar range [32]. Not being in the scope for this study, it can be envisioned that fitting of such experimental IC formation data will be of benefit for setting up adverse events evading drug dosing regimens. A direct analysis of the ICs at physiological concentrations without modification of the involved components is a breakthrough and although requiring further refinement, i.e., increasing the precision for the quantification of individual IC species, the new approach we used in this work will significantly change analytics in the field of (immune) complex formation.

A critical aspect in the assessment and characterization of ICs is the suitability and performance of the analytical methods. Dependent on the scientific question, a specific method may be more suitable than another. SEC, as a reliable high-throughput method, has superior resolution in the lower M_W_ range and is suitable to discriminate between antigen:biotherapeutic complexes from small immune complexes containing ADA(s). However, due to the limited resolution at higher M_W_ range, the methodology is not suitable to provide sufficient insight into the characteristics of larger ICs above 500 kDa, without extensive adaptations. Additional general challenges of SEC to assess complex formation are related to material dilution on the column, interaction of the non-covalent complexes with the stationary phase with the risk of unwanted complex dissociation, incomplete mass recovery [33], and protein complex dissociation provoked by the running buffer. Especially the latter was observed for other biotherapeutis as the root cause for the abrogation of IC formation (data not shown). Therefore, the analysis of immune complexes in clinically relevant matrices, at least as a control strategy, is highly advisable. SV-AUC and mass photometry represent methods that overcome challenges described for SEC by direct analysis of samples in the matrix of interest. In contrast to SEC, these methods have the advantage of improved resolution power at higher M_W_ range (>500 kDa).

According to the scope of the study, the implemented SV-AUC method allowed a reliable and reproducible assessment of formed IC species. Where required, newer developments in the field might be of help for in-depth characterization of detected individual protein-complex species. Multispeed SV-AUC enables focusing on certain areas of the size-distribution, i.e., clearly resolving non-complexed and complexed species of various sizes. Simulations of the impact, which the different speed profiles have on the separation of individual species, led to the development of new data evaluation tools for AUC as implemented in Ultrascan [34] or Sedanal [35]. Besides conventional single-wavelength experiments, several multi-wavelength AUC approaches have been developed like in Ultrascan [36], Sedanal [37], and sedfit/sedphat [38,39]. For spectrally similar proteins, an improved version with additional constraints was developed [40]. These methods rely on the spectral discrimination between components of a solution mixture by acquiring AUC data at two or more wavelengths or even collecting full spectra at each radial position of an AUC experiment. Such analyses allow exact confirmation of stoichiometry between components of detected complex species. This approach was unfortunately not feasible in the current study, due to the virtual absence of spectral differences between the biologic and ABAs.

Very good reproducibility and high sensitivity are observed for SEC as the main workhorse of the analytical labs. Higher, but still acceptable variability is observed with SV-AUC and mass photometry. Mass photometry is a newer technology successfully applied in the immunological field [41]. In our studies on the formation of immune complexes, however, extensive adaptations were required. This included pre-evaluation of sample preparation work to avoid material loss by adsorption and project-specific adaptations introducing appropriate control strategies and result-averaging from repeated sample analyses. The latter is a must to obtain reliable (semi-)quantitative data regarding relative abundances of individual IC species. An additional minor limitation of mass photometry is the lower limit of detection for smaller- sized molecules below ~70 kDa. For the compositions of proteins tested in our study, the assessment of free components and antigen:biologic complexes was not feasible. Ultimately, it must be emphasized that none of the used methods on their own is sufficient to analyze all aspects involved in the process of immune complex formation, but are complementary to a successfully and balanced characterization.

The biggest challenge for the analysis and characterization of ICs by available analytical tools is posed by the complexity of biofluid-matrices, i.e., the detection of immune complexes and associated free components in serum or plasma background. Multiple analytical techniques like MALS and mass photometry are generally not suitable from the technical perspective. Recent publications are indicating DLS to be an appropriate tool, capable to assess ICs in buffered systems and in serum [42,43]. These sophisticated and customized DLS studies enable the detection of relative abundances of individual IC species, but require special equipment, tailor-made procedures, and high-end expert control strategies, including custom made algorithms for correct data interpretation. Commercially available DLS solutions generally used in pharmaceutical industry are not suitable for the characterization and quantification of immune complexes. SEC and SV-AUC require adaptations in the methodology to assess ICs in serum [44], for example by fluorescence labeling of components or decreasing the concentration of the serum. The first option might impact binding interfaces between the components and impair the formation of ICs. The second option might not mimic the physiological situation correctly and underestimate the impact of the serum components on IC formation. For the chosen model system described in this study, no interference from serum components was detected under diluted serum conditions, suggesting that a severe interaction between serum and IC components can be excluded. Nevertheless, the differential assessment approach we describe is an advantageous tool in the quantitative analytical portfolio. It can be used to identify time-dependent changes e.g., comparing pre- and post-dosing patient-derived samples.

Predictive in silico [11,45] and other immunogenicity-potential assessment tools like the MAPPs assay [12] can provide valuable insights for the required immunogenicity risk assessments [14] and the selection process to define lead candidates with the lowest immunogenic potential during developability assessment phases [10]. The analytical tool-box presented in this manuscript, designed to shed light on factors influencing IC formation, is a well suited state-of-the-art screening and prediction instrument, which can be used in research organizations to obtain additional insights into the behavior of biotherapeutic lead candidates in a simulated scenario of immunogenicity. Moreover, the same tool-box is useful for assessing degradation or aggregation of biotherapeutics and their potential impact on the formation of ICs. Protein degradation is specifically becoming a challenge for new biotherapeutic formats containing non-immunoglobulin fold domains [20]. The impact of shelf-life-related degradation on IC formation may also be assessed via this tool box, using material from forced-degradation or accelerated stability studies in comparison to non-stressed material. Alternatively, the assessment of IC formation using degraded biologics material from incubation in biomatrices like serum could be an attractive option. Due to their extended half-life, many biologics are known to experience modifications from being exposed to elevated temperatures and to serum components [46].

Besides these immunogenicity liability de-risking activities for early phase drug candidates, the results of surrogate in-vitro IC-formation studies will have supportive and guiding character for clinical studies. The generated data from such in-vitro experiments can be considered as pre-work to ensure the suitability of utilized analytical methods for clinical immunogenicity assessment studies [30,47]. With adaptations, the presented analytical tool-box has the potential to be used in clinical studies to assess the abundance of ICs, in addition to the routinely assessed free ADA and free biotherapeutic contents [48]. Ultimately, modelling of the generated in-vitro IC characterization data might be used to guide clinical study designs aiming for so called immunomodulatory regimens to reduce the risk of adverse immunologic response events for patients, as successfully established for rituximab in severe haemophilia A [49].

## Data Availability

Due to internal confidentiality restrictions, raw data and compound material will be not shared. Further information about compounds and used methods will remain confidential.

## Supplementary Materials

The following are available online at https://www.mdpi.com/article/10.3390/pharmaceutics14061254/s1, Figure S1: Assessment of complex formation between antigen, biologic and monoclonal anti-biologic antibodies (mABAs) by SV-AUC, Figure S2: Assessment of complex formation between antigen, biologic and monoclonal anti-biologic antibodies (mABAs) by DLS, Figure S3: Assessment of immune complex formation between biologic:pABA at a molar ratio of 1:1 by mass photometry, Figure S4. Replicate mass photometry analysis of a sample containing immune complexes.
