# Supplementary material for: Pre-Clinical In-Vitro Studies on Parameters Governing Immune Complex Formation
Source: Pharmaceutics. 2022 Jun 13;14(6):1254. doi: 10.3390/pharmaceutics14061254 (PMC9227392; doi:10.3390/pharmaceutics14061254)
Supplement: Supplementary file 1 [file pharmaceutics-14-01254-s001.zip › pharmaceutics-1732727-supplementary.pdf]

Supplementary Materials: Pre-clinical *in-vitro* studies on parameters governing immune complex formation

Marie Fichter, Gesa Richter, Alexander Bepperling and Paul Wassmann

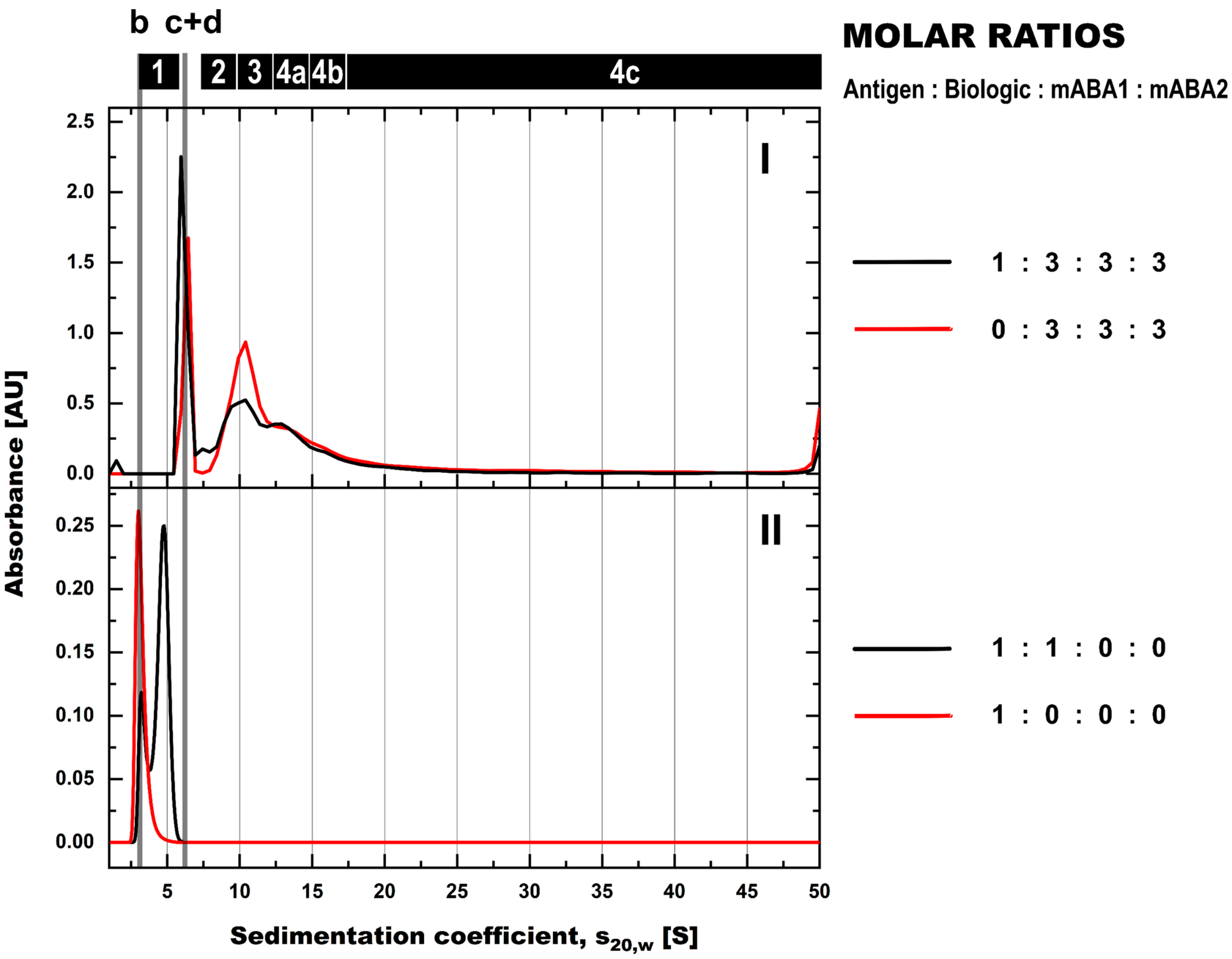

Figure S1. Assessment of complex formation between antigen, biologic and monoclonal anti-biologic antibodies (mABAs) by SV-AUC. Panel (I) is showing profiles of compositions between biologic, mABA1 and mABA2 in an equimolar ratio (red trace), and between antigen:biologic:mABA1:mABA2 in a molar ratio 1:3:3:3 (black trace). Panel (II) is showing profiles of the antigen (red trace) and of an equimolar composition between the antigen and biologic. Marker: (b) free antigen, (c) free mABA1, (d) free mABA2, (1) antigen:biologic complexes, (2) immune complexes with a single ABA incorporated, (3) immune complexes with two ABAs incorporated, (4a) immune complexes with three ABAs incorporated, (4b) immune complexes with four ABAs incorporated, (4c) immune complexes with more than four ABAs incorporated.

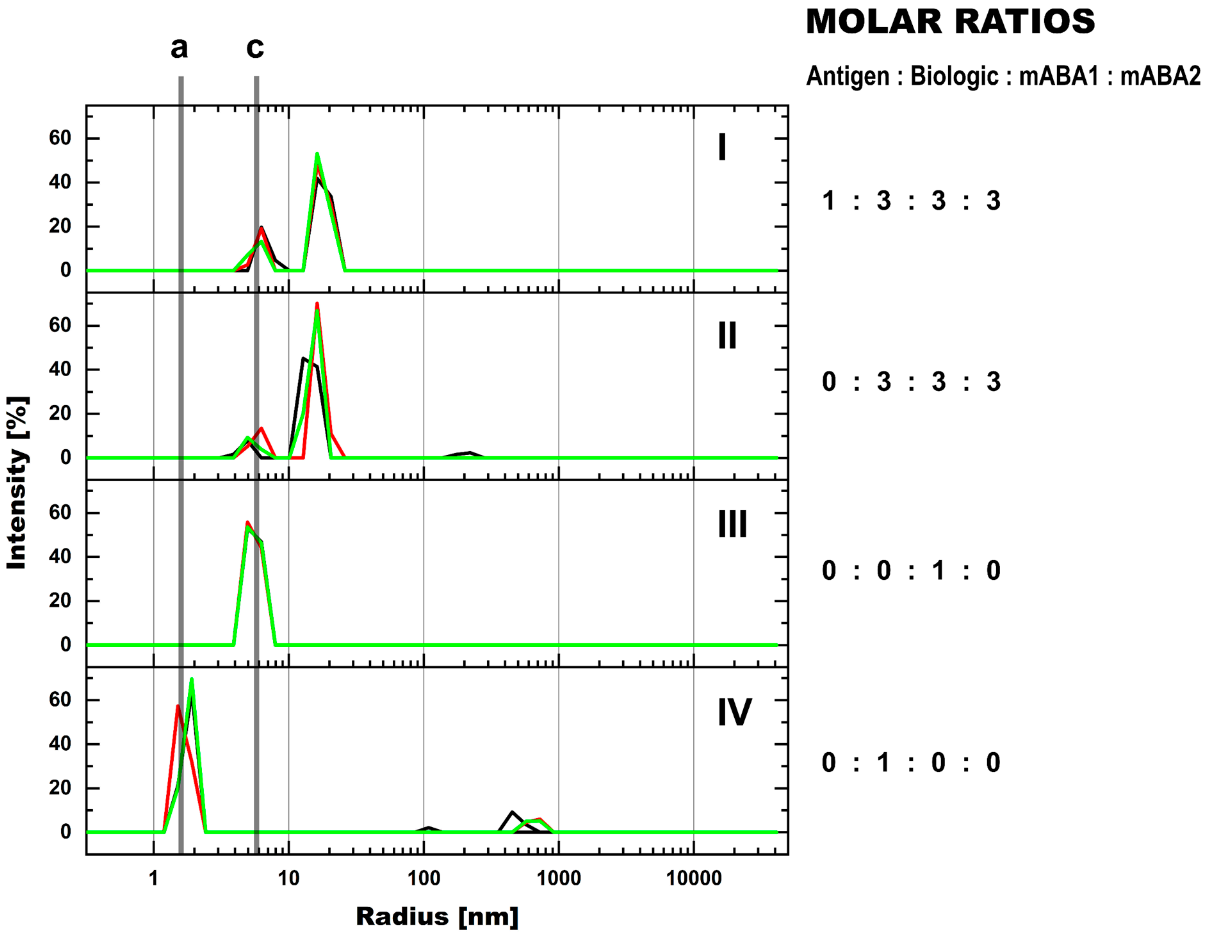

Figure S2. Assessment of complex formation between antigen, biologic and monoclonal anti-biologic antibodies (mABAs) by DLS. Compositions are shown between antigen:biologic:mABA1:mABA2 in a molar ratio 1:3:3:3 on panel (I), and between biologic, mABA1 and mABA2 in an equimolar ratio on panel (II). DLS profiles of control molecules, i.e. mABA1 and biologic, are shown on panels (III) and (IV), respectively. All samples were analyzed in triplicates and are represented by red, green and black traces. Marker: (a) free biologic, (c) free mABA1. mABA1.

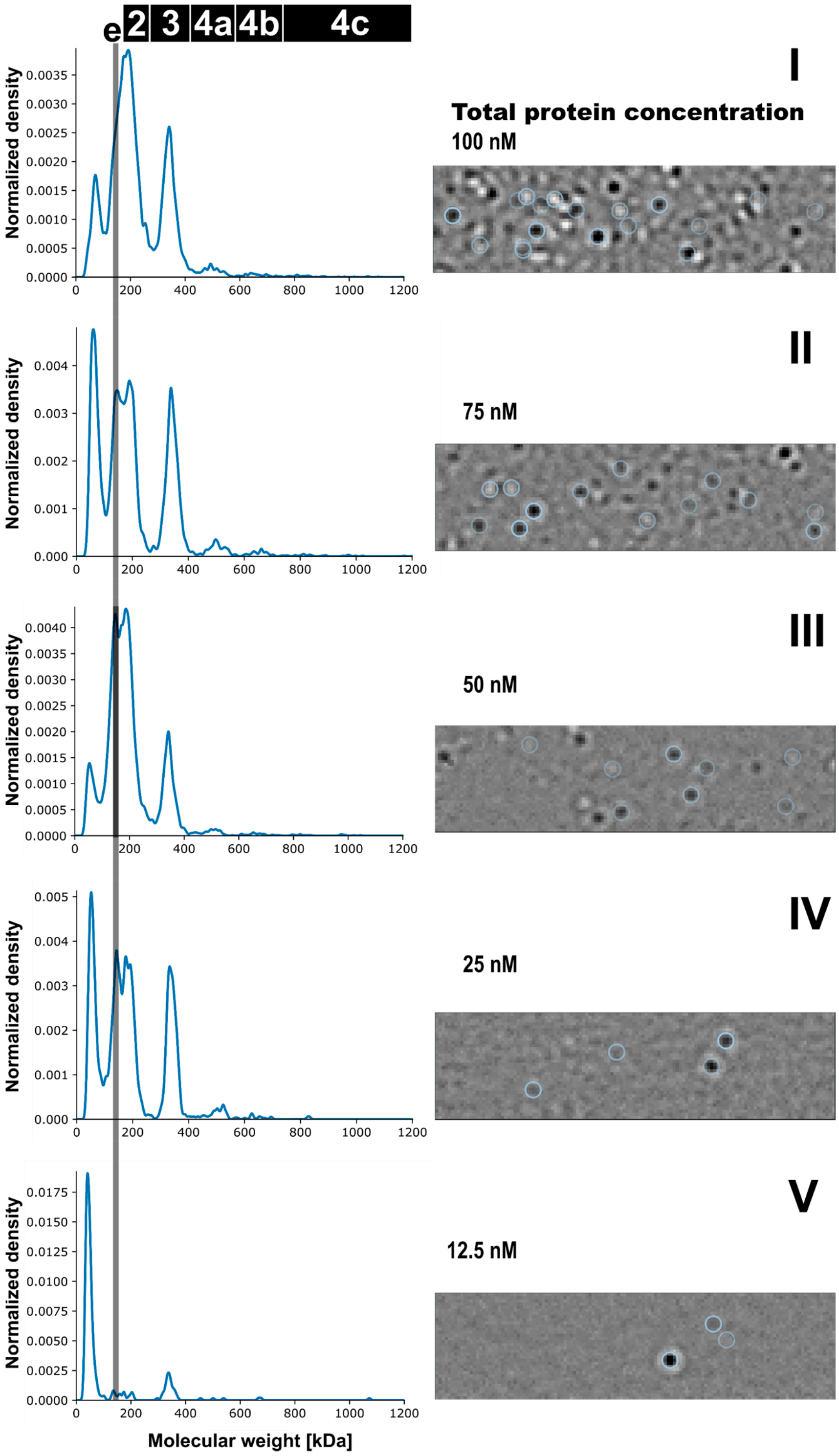

Figure S3. Assessment of immune complex formation between biologic:pABA at a molar ratio of 1:1 by mass photometry. Panels (**I**) to (**V**) show the outcome of experiments performed at total protein concentrations of 100, 75, 50, 25 and 12.5 nM, respectively. MP kernel density estimate profiles (left column) and selected MP frames (right column) are shown for the performed analyses. Blue circles in MP frames indicate detected particles, which were recognized by the software. At the highest tested concentration of 100 nM a significant overlap of the signals can be observed, which generates result artefacts. At the lowest tested concentration of 12.5 nM an insufficient number of events is detected, leading to underrepresentation of low abundancy species. Analysis at a total protein concentration of 50 nM provides well balanced particle detection for the present biological system. Marker: (e) free pABA, (2) immune complexes with incorporation of a single ABA, (3) immune complexes with two ABAs incorporated, (4a) immune complexes with three ABAs incorporated, (4b) immune complexes with four ABAs incorporated, (4c) immune complexes with more than four ABAs incorporated.

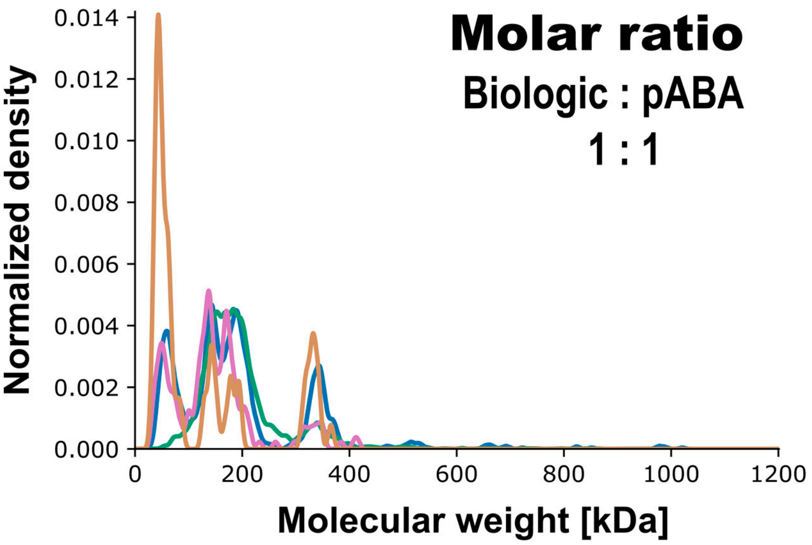

Figure S4. Replicate mass photometry analysis of a sample containing immune complexes. Four independent MP readouts are shown for a single sample. The sample with the total protein concentration of 50 nM is composed of biologic:pABA at a molar ratio of 1:1.
